# Skeletal Manifestations, Bone Pain, and BMD Changes in Albanian Type 1 Gaucher Patients Treated with Taliglucerase Alfa

**DOI:** 10.1155/2023/3254533

**Published:** 2023-12-04

**Authors:** Virtut Velmishi, Erjon Troja, Marjeta Tanka, Donjeta Bali, Ermira Dervishi, Afërdita Tako, Laurant Kollcaku, Paskal Cullufi

**Affiliations:** ^1^University of Medicine, Pediatric Department, Gaucher Unit, “Mother Teresa Hospital”, Tirana, Albania; ^2^University of Medicine, Faculty of Medicine, Department of Pharmacy, Tirana, Albania; ^3^University of Medicine, Pediatric Department, “Mother Teresa Hospital”, Tirana, Albania

## Abstract

Gaucher disease is a rare, genetic lysosomal disorder leading to lipid accumulation and dysfunctions in multiple organs. Bone involvement is one of the most prevalent aspects of Gaucher disease. Pain, disability, and reduced quality of life remain the most frequent characteristics of bone involvement in Gaucher patients. *Patients and Method*. In this study, we will take into consideration data from 24 patients diagnosed with type 1 Gaucher disease. We followed them closely for six years in progress. At baseline, all patients started therapy with taliglucerase alfa at a mean dosage of 45 UI/kg; later, during the study, two of them switched their cure toward velaglucerase alfa. Before baseline evaluations, 12 patients had been treated with imiglucerase at variable duration times. At baseline, we performed an X-ray of long bones and the spine, and each year, different standard assessments were performed, such as bone pain, MRI of the vertebral spine and pelvis, and DEXA for bone density. Four patients left the study for various reasons, two of them at baseline and two others during year 3 (FU3). *Results*. At baseline, we had 8 children and 16 adults. The average age was 28.7 ± 16.5 SD years. The most frequent skeletal manifestations in our patients were reduction of tibial femoral space (40%), osteonecrosis (36%), and body vertebral reduction (32%). At baseline, 15 patients presented with bone pain to different degrees. Over the years, bone pain in our patients had a gradual improvement. The most dramatic bone pain improvement was seen in a patient who presented bone crises. Another impressive finding was a significant BMD improvement during six years of treatment. Our study showed a significant improvement in BMD comparing FU5 and baseline values (*p* = 0.0007). Especially children demonstrated a significant improvement in BMD (*p* = 0.00061) compared to adults (*p* = 0.3673). Mean BMD change was more indicative in switched patients (*p* = 0.0142) compared to naïve patients (*p* = 0.147). *Conclusions*. Skeletal manifestations are very different in Gaucher type 1 patients. In our study, as a result of long-term evaluations, it was noticed that the most frequent skeletal manifestation was a reduction of tibiofemoral space. Bone pain has gradually improved in all patients. Also, BMD values have been enhanced over six years of treatment, especially in children.

## 1. Introduction

Gaucher disease is one of the most common lysosomal diseases [1]. It is characterized by a deficiency of acid *β* glucosidase that impairs glucosylceramide's catabolism, leading to glycolipid accumulation. Gaucher cells are seen in most tissues, but the spleen, liver, and bone marrow are some of the most affected. Consequently, patients with GD manifest visceromegaly, cytopenia, and diverse skeletal lesions [2, 3]. Skeletal manifestations are the primary cause of pain, disability, and reduced quality of life in patients with Gaucher disease. GD affects the bone marrow and mineralized components of the bone. Changes include bone marrow infiltration, modeling, and bone remodeling, resulting in osteopenia-osteoporosis, cortical thinning, lytic lesions, osteonecrosis, and fragility fractures [4–6]. Our study will focus on bone changes in our patients diagnosed with GD.

## 2. Patients and Method

**Figure 1:**
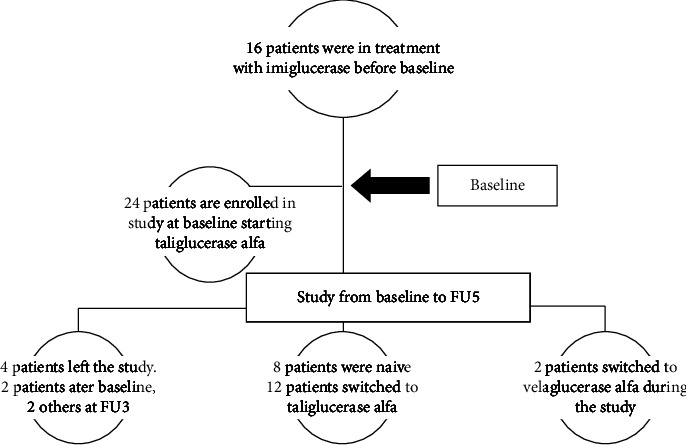
Distribution of our patients before baseline, at baseline, and during the study.

**Figure 2:**
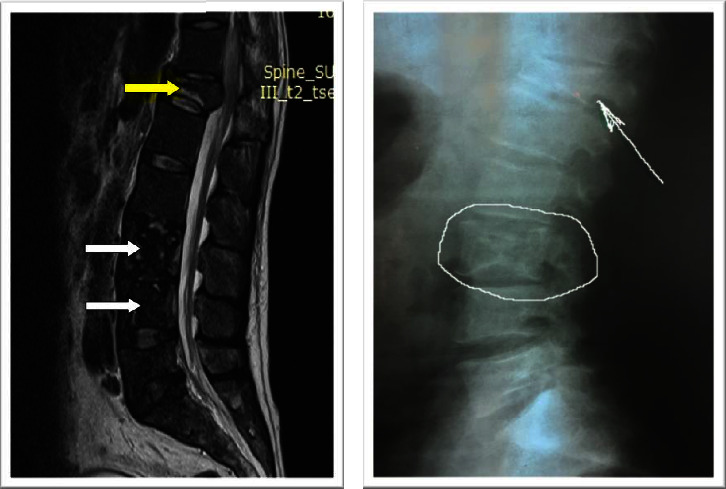
(a) Osteonecrosis of vertebral bodies L3 and L4 (white arrows) and compressive fracture of Th12 (yellow arrow) on MRI. (b) Lateral X-ray of the lumbar spine showing the destruction of Th 12 (arrow) and L3 (circle).

**Figure 3:**
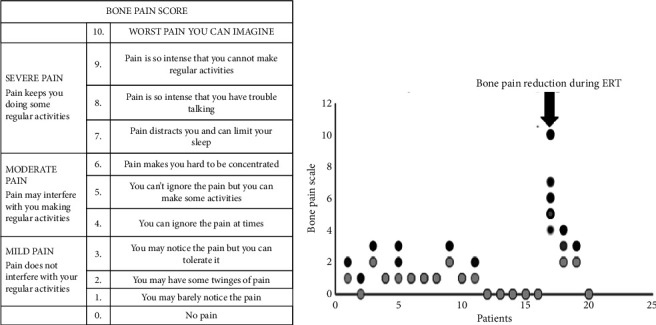
Numerical pain score which is adapted for bone pain (a) and improvement of bone pain during six years (b). The darkest points showed the bone pain scale at baseline, and the lightest points showed recent FU.

This review study uses collected data from 24 patients diagnosed with Gaucher disease type 1, treated and followed at our center during many years of healthcare activities (signed informed consent is taken for each patient, related to data disclosure). For pediatric patients, specific signed ICF parental consent has also been performed. We have completed the study-described activities at our Gaucher Unit, the only metabolic center in Albania, next to the Pediatric Department at Mother Teresa Hospital. The scientific research data were obtained from 2015-2016 (first baseline evaluations) to 2020-2021 (follow-up-year 5). These patients have been followed up minimally for six years and further. At the start (baseline evaluations), we had 8 children and 16 adult subjects. The average age was 28.7 years ± 16.5 SD. The maximum age was 72 years old, and the minimum age was 7 years old. In the beginning, all patients started therapy with taliglucerase alfa (24 patients), and two of them switched to velaglucerase alfa because one patient presented adverse events and the other changed due to administrative issues. Before baseline, 12 patients were under therapy with imiglucerase at variable times (Figure 1). The most frequent genotype (19 patients) was p. Asn409Ser: p. His294Gln/pAsp448His or N370 S:H255Q/D 409H according to old nomenclature (Table 1). The mean dosage of taliglucerase alfa during the study was approximately 45 UI/kg. Switched patients to velaglucerase alfa have been at the exact dosage as before. We have performed for each patient a plain X-ray of the long bones and spine (baseline), an MRI of the abdomen and pelvis (every year), DEXA for L1-L4 and the right and left hip (every year), lab analyses (every year), and a bone pain assessment (every year). All radiological images obtained were interpreted by one radiologist with longtime experience in bone assessment of GD patients. In this study, we are focused only on bone changes. The main topics of interest were as follows:

1. Evaluation of skeletal manifestations: For each patient, we have evaluated X-ray and MRI images. The analyses included all 24 patients' data and images at baseline. Two patients with bone crises and severe bone changes, such as compressive fractures and vertebral osteonecrosis, left the country after baseline. They were brother and sister, respectively, 43 and 45 years old, and both were splenectomized. We have presented a lateral X-ray of the spine and an MRI of the same patient (brother). It was noticed that there was osteonecrosis of vertebral bodies L3 and L4 and a compressive fracture of Th12 (Figure 2). Two other patients left the study during year three (FU3). One left the country, and the other had a severe adverse event. He refused to continue the therapy even though we proposed switching him to another ERT alternative.
2. Evaluation of bone pain: Every year, each patient has been assessed for bone pain based on a bone pain score adapted by myhealth.alberta.ca (https://myhealth.alberta.ca/health/Pages/conditions) as a numerical pain scale (Figure 3). All the results were registered in points. The darkest colored points showed the bone pain scale at baseline, and the lightest points showed recent FU. In some patients, you may see only one point, meaning that the bone pain scale remains at the same level. We have signed the score for each patient during the six years, and the data have been reviewed at the end of the study.
3. Assessment of bone mineral density: Yearly, we have evaluated every value collected of DEXA (BMD) L1-L4 of the lumbar spine for each patient (using a Dexa-Stratos densitometer). All data were processed using the MS Excel program over a period of six years. We have analyzed data on BMD evaluating mean BMD for each year of study. We compared the mean BMD between FU5 and baseline. Also, we calculated mean BMD values between children/adults and naïve/switched patients.

## 3. Results and Discussion

### 3.1. Skeletal Manifestations

**Figure 4:**
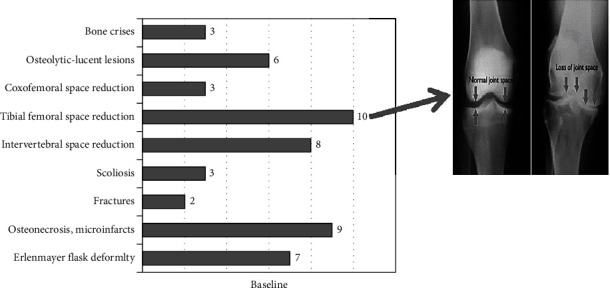
Skeletal manifestations in GD patients at baseline.

Skeletal manifestations can vary among individuals with Gaucher disease. Our first goal was to find the most frequent skeletal manifestations in our group of patients. We have analyzed the radiological data for every patient. The most common finding at baseline was a reduction of tibial femoral space (Figure 4). Two of our patients who presented with bone crises left the study. They both (siblings) presented osteonecrosis and compressive fracture of vertebrae (Figure 2). The tibial femoral space reduction finding was unexpected to us as the most common radiological finding in our patients. We thought Erlenmeyer flask deformity could have been one of the most frequent findings, but in our group, the result was only 28%. Erlenmeyer flask deformity occurs before puberty, develops progressively, and is present in 80% of adults with Gaucher disease [7]. Lytic lesions are also very frequent in GD patients. The bone has a “worm-eaten” aspect with a radiologically rarefied cortex and dentate endosteum [7]. We found only six patients with osteolytic-lucent lesions (6%). Osteonecrosis remains the most relevant and invalidating skeletal manifestation secondary to bone infarction. The most affected areas are the femoral head, proximal humerus, and vertebral bodies [8]. Once established, it is irreversible [9]. In our study, we found osteonecrosis in 36% of our patients, the second most frequent finding in our group of patients. At baseline, we had three patients (12%) who presented with bone crises, but two of them left the study. A bone crisis is an acute episode of severe pain with periosteal elevation on radiographs that may occur in patients with mild or severe disease. Of a total of 2004 patients enrolled in the International Gaucher Group registry from 1991 to 2001, between 76% and 94% of those with GD type 1 had a radiological manifestation of bone disease, including marrow infiltration, Erlenmeyer flask deformity, or osteonecrosis [7]. Bone disease is often asymptomatic and may sometimes progress despite effective treatment [10]. Less frequent findings in our patients were fractures, scoliosis, and reduction of the coxofemoral space. At the end of the study, we repeated an X-ray of the long bones and spine for every patient which did not reveal any essential changes.

### 3.2. Bone Pain Evaluation

Bone pain is a common symptom of Gaucher disease and is often one of the earliest signs. The accumulation of fat cells in the bone marrow can cause bone thinning, weakening, and pain. This pain typically affects long bones, such as the thigh and arms, but can also occur in other bones. For each patient, we assessed bone pain. In this context, we have used a bone pain score adapted from the numerical pain rating scale of myhealth.alberta.ca (https://myhealth.alberta.ca/Health/Pages/conditions). All the results were registered in points. The darkest colored points showed the bone pain scale at baseline, and the lightest points showed recent FU. You may see only one point in some patients, meaning that the bone pain scale remains at the same level (Figure 3). From 20 patients in total, we had 15 patients at baseline who presented bone pain in different degrees. Reduction in the bone pain scale is evident in all patients. Bone pain is generally variable, ranging from dull, achy, and nonspecific to intense or localized.

In contrast to bone pain, bone crises are accompanied by elevated white cells and fever. The debilitation may last over three days, usually requiring immobilization and narcotics for pain relief [11]. Bone crises are often followed by necrosis and fractures [12]. Metabolic stress, such as pregnancy, may trigger bone crises [12–14]. Bone crises in GD may occasionally be confused with osteomyelitis [15]. One of our patients was diagnosed with GD after hospitalization in the infectious disease ward. He had a prolonged fever, hepatosplenomegaly, and bone crises. Initially suspected for osteomyelitis, but after bone marrow aspiration, Gaucher cells were noticed. This patient showed a dramatic reduction in the bone pain scale over the course of six years (see the black arrow in Figure 3). At the end of the study, his bone pain scale was 4. Of the other 14 patients who presented with bone pain at baseline, at the end of the study, four patients had only mild pain (pain scale = 2), eight patients had minimal bone pain (pain scale = 1), and two patients had no bone pain (pain scale = 0). Another element distinguishing a bone crisis from osteomyelitis is the absence of a left shift in the white cells and negative blood cultures [16]. Two other siblings who presented with bone crises are brother and sister, both splenectomized. Splenectomy, which was frequently carried out in GD patients to control thrombocytopenia, is thought to exacerbate the risk of osteonecrosis [17–19]. Splenectomized patients have more inflammatory activity and severe bone disease than nonsplenectomized patients [17]. There is an ongoing debate between relational splenectomy and osteonecrosis. Actually, it is considered a causal relationship [20]. Therefore, splenectomy should be avoided to shun the development of osteonecrosis [21].

### 3.3. Bone Densitometry Assessment

Bone mineral densitometry is crucial in assessing the skeletal involvement in Gaucher disease. By measuring bone mineral density, healthcare professionals can identify bone loss and determine the risk of fractures. Osteopenia is quite universal in both children and adults with GD type 1. It can be localized or diffused and is associated with an increased risk of pathological fracture [22]. ICGG Gaucher registry data indicate that 55% of all patients have investigator-defined osteopenia [23].

**Figure 5:**
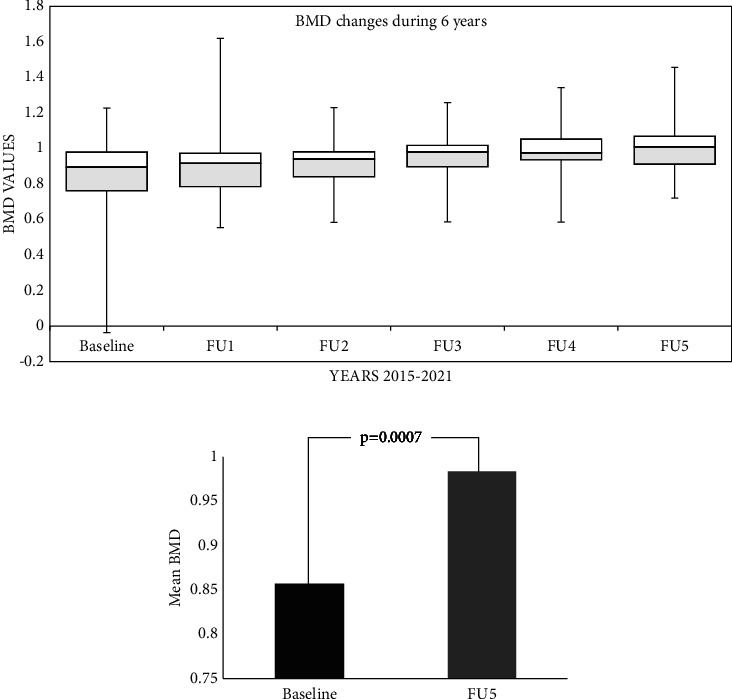
The difference in BMD changes between baseline and FU5.

In our study, we evaluated BMD every year for every patient using a Dexa-Stratos densitometer. DEXA of the lumbar spine L1-L4 and DEXA of the right and left femoral hip were performed as a procedure. We have analyzed data on BMD for the lumbar spine (Figure 5). As you may see in Figure 5(a), a progressive improvement of BMD is present each year during the six years. The difference between baseline and FU5 is very significant, showing a value of *P* = 0.0007 using the Student *T*-test (Figure 5(b)). A *Z*-score of −1 or less in GD patients increases the risk of a fragility fracture by a factor of 5 [24, 25], which is a significantly higher estimate than the commonly associated osteoporotic fracture in population studies [26]. In adults with GD, the average BMD *Z*-score is approximately −1, but with a wide distribution [27].

In our study of adult patients at baseline, the average BMD value was 1.024, and in our children with GD, it was 0.702. At FU5, we found an average value of BMD in children, 0.985, and in adults, 1.036.

**Figure 6:**
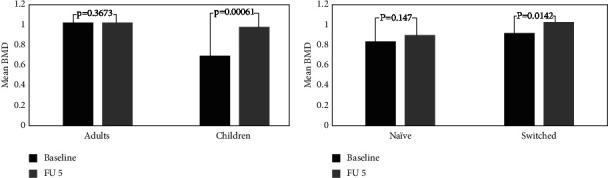
BMD values comparing children with adults (a) and naïve patients with switched patients (b).

At the same time, calculating the changes between baseline values of BMD and FU5 using *T*-test (Figure 6(a)) shows a significant improvement of BMD in children (*p* = 0.00061), while it was found that there was no significant improvement of BMD in adult patients (*p* = 0.36733). Another evaluation was comparing switched patients to naïve patients using the same method. In switched patients, the average values of BMD baseline/FU5 were 0.921 and 1.037, respectively (Figure 6(b)). In the naïve patient group, the average values of BMD were 0.839 and 0.904. We compared the two groups using the *T*-test, which showed a significant improvement of BMD in switched patients (*p* = 0.0142) compared to naïve patients (*p* = 0.147). This result may be explained by the high number of children present in the switched patient group.

A BMD *Z*-score of less than −2 occurs early in GD patients and may occur by the age of 5 years [27]. Patients who underwent splenectomy have a lower BMD than nonsplenectomized patients [28], but still, it is not clear whether this is simply a reflection of more significant disease severity or an effect of the splenectomy. In our study at baseline, we had two patients splenectomized with severe bone involvement and bone crises, but unfortunately, they left the study earlier, and we could not complete their full follow-up activities.

The poor prognosis of bone health in Gaucher patients depends on three factors: a low dosage of enzyme therapy (generally under 45 UI/kg) and no adequate adherence, splenectomy, and at last, delayed diagnosis. In our groups, two factors were excluded: dosage and adherence were adequate, and splenectomized patients left the study; the most critical factor remains the time of diagnosis which was variable from months to years in our patients. Ultimately, we would like to emphasize that the cornerstone of bone health in Gaucher patients remains early diagnosis and adequate treatment in nonsplenectomized individuals.

The main limitation of this study is the small number of patients, but this is normal for a rare disease pathology such as Gaucher disease, also considering the overall Albanian population. The loss to follow-up number of patients is also low (just four, lowering the total number of evaluated patients from 24 to 20).

## 4. Conclusions

Skeletal manifestations are very different in Gaucher type 1 patients. In our study, as a result of long-term evaluations, it was noticed that the most frequent skeletal manifestation was a reduction of tibiofemoral space. Bone pain has gradually improved in all patients. Also, BMD values have been enhanced over six years of treatment, especially in children.

## Figures and Tables

**Table 1:** General data of our patients.

Number of patients at baseline	24

Number of patients at the end of study	20

Gender (F-M)	7/13 (end of the study)

Genotype	19/p.Asn409Ser:p.His294Gln/pAsp448His
	2/p.Asn409Ser:pArg 502His
	1/p.Asn409Ser:pSer146Leu
	1/p.Asn409Ser:p/Arg86^*∗*^
	1/p. Asn409Ser: p/LeuProfs^*∗*^4: Arg87Trp

Age at baseline	28.7 ± 16.5 (min age: 7 y, max age: 72 y)

Children (under 18 y)/adults	8/16

Naïve patients	8 patients

Switched patients	12 patients switched at baseline (imiglucerase to taliglucerase)
	2 patients switched during FU (taliglucerase to velaglucerase)

Splenectomized	2

## Data Availability

The data supporting the findings of this study are included within the article.
